# Deep Active Learning for Automatic Mitotic Cell Detection on HEp-2 Specimen Medical Images

**DOI:** 10.3390/diagnostics13081416

**Published:** 2023-04-14

**Authors:** Asaad Anaam, Mugahed A. Al-antari, Jamil Hussain, Nagwan Abdel Samee, Maali Alabdulhafith, Akio Gofuku

**Affiliations:** 1 Graduate School of Interdisciplinary Science and Engineering in Health Systems, Okayama University, Okayama 700-8530, Japan; 2 Department of Artificial Intelligence, College of Software & Convergence Technology, Daeyang AI Center, Sejong University, Seoul 05006, Republic of Korea; 3 Department of Data Science, College of Software & Convergence Technology, Daeyang AI Center, Sejong University, Seoul 05006, Republic of Korea; 4 Department of Information Technology, College of Computer and Information Sciences, Princess Nourah bint Abdulrahman University, P.O. Box 84428, Riyadh 11671, Saudi Arabia

**Keywords:** medical HEp-2 specimen images, HEp-2 mitotic cell detection, deep active learning (DAL), automatic data annotation, computer-aided detection (CAD)

## Abstract

Identifying Human Epithelial Type 2 (HEp-2) mitotic cells is a crucial procedure in anti-nuclear antibodies (ANAs) testing, which is the standard protocol for detecting connective tissue diseases (CTD). Due to the low throughput and labor-subjectivity of the ANAs’ manual screening test, there is a need to develop a reliable HEp-2 computer-aided diagnosis (CAD) system. The automatic detection of mitotic cells from the microscopic HEp-2 specimen images is an essential step to support the diagnosis process and enhance the throughput of this test. This work proposes a deep active learning (DAL) approach to overcoming the cell labeling challenge. Moreover, deep learning detectors are tailored to automatically identify the mitotic cells directly in the entire microscopic HEp-2 specimen images, avoiding the segmentation step. The proposed framework is validated using the I3A Task-2 dataset over 5-fold cross-validation trials. Using the YOLO predictor, promising mitotic cell prediction results are achieved with an average of 90.011% recall, 88.307% precision, and 81.531% mAP. Whereas, average scores of 86.986% recall, 85.282% precision, and 78.506% mAP are obtained using the Faster R-CNN predictor. Employing the DAL method over four labeling rounds effectively enhances the accuracy of the data annotation, and hence, improves the prediction performance. The proposed framework could be practically applicable to support medical personnel in making rapid and accurate decisions about the mitotic cells’ existence.

## 1. Introduction

Connective tissue diseases (CTD) are autoimmune disorders initiated by generating abnormal autoantibodies that act against nuclear and cytoplasmic antigens and so-called anti-nuclear antibodies (ANAs). ANAs affect body tissues, causing chronic inflammatory disorders such as systemic lupus erythematosus, rheumatoid arthritis, systemic sclerosis, Sjögren’s syndrome, and idiopathic inflammatory myopathies [1]. Diagnosing such diseases involves identifying and measuring the corresponding ANAs in the patients’ serum [1]. In this regard, the indirect immunofluorescence using human epithelial type-2 cells (IIF HEp-2) substrates is considered the standard serological protocol for ANAs testing [2].

In the IIF HEp-2 protocol, several ANAs appear as distinct HEp-2 cell fluorescence patterns, and each one is associated with a particular disorder [3,4]. The IIF HEp-2 protocol involves several visual analysis steps at the fluorescence microscope, including determining the positivity strength of the specimen (slide), recognizing the HEp-2 cells in the mitotic phase, and then classifying the staining patterns of interphase cells to identify the type of ANAs [4]. As it requires several steps for slide preparation and visual pattern interpretation, the IIF HEp-2 manual protocol is a subjective and semi-quantitative method that heavily depends on the expertise of the practitioner, which increases the diagnosing variability across observers or laboratories. In addition, the preparation time requirement reduces the throughput of this test. Accordingly, the limitations of the manual IIF HEp-2 protocol have motivated recent interest in developing reliable computer-aided diagnosis (CAD) systems to support the diagnostic procedure [4,5]. However, a small portion of this interest is directed to approaching the task of mitotic cell identification even though it is a crucial step in the IIF HEp-2 diagnostic procedure, and hence, for developing a feasible HEp-2 CAD system [4]. Recently, the CAD system based on deep learning has gotten much attention for automating the prediction process with a massive number of extracted deep features, improving detection performance, and reducing user intervention during the training process [6,7].

**Figure 1:**
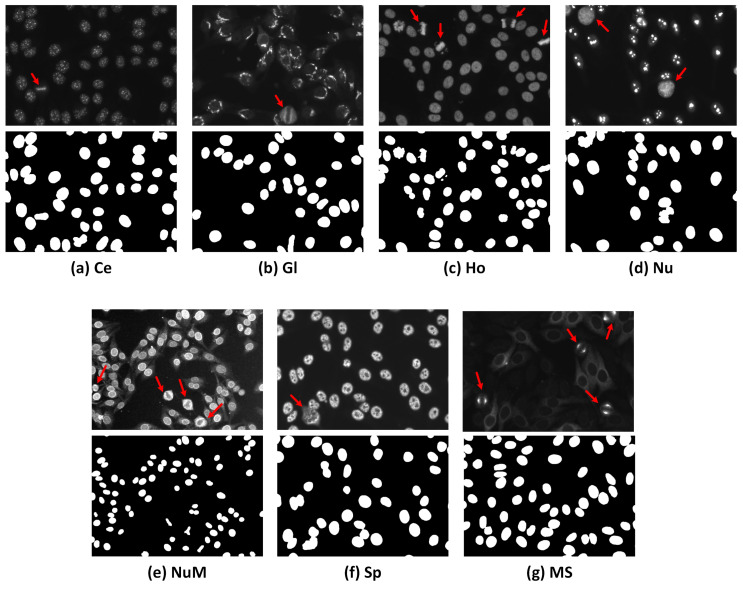
Examples of specimen images from the I3A Task-2 dataset. The first row shows the FITC images, while the second row shows their corresponding DAPI channel binary masks. Classes annotated as: (**a**) Centromere (Ce); (**b**) Golgi (Gl); (**c**) Homogeneous (Ho); (**d**) Nucleolar (Nu); (**e**) Nuclear Membrane (NuM); (**f**) Speckled (Sp); and (**g**) Mitotic Spindle (MS). The red arrows indicate the mitotic cells appearing in the HEp-2 specimen images.

HEp-2 cell patterns could be classified based on the cell cycle phases into two broad categories, i.e., mitotic patterns (cell under division phase) and interphase patterns (non-mitotic phase). The HEp-2 cells undergo the mitotic phase through 10∼15% of their entire lifecycle, causing their appearance in the HEp-2 specimen images to be very rare compared to the interphase cell patterns [4]. During the mitotic phase, HEp-2 cells exhibit distinct patterns as the cells’ DNA has different concentrations and distributions compared to the interphase counterpart [8,9,10,11]. Figure 1 shows examples of mitotic cells occurring in different HEp-2 specimen interphase patterns and their corresponding binary masks. Mitotic cell identification is clinically important for validating the correctness of the specimen’s preparation procedures and providing supportive information for discriminating between some ambiguous interphase patterns in the mixed patterns specimen images. This is because some mitotic patterns match certain interphase types [4].

HEp-2 mitotic cell detection using HEp-2 specimen images has barely been studied in the literature of HEp-2 image analysis [4]. Typically, the vast majority of these works addressed this task as a binary classification problem between the minority mitotic class against the remaining majority interphase class in the cell-level images [9,10,11]. However, addressing this task in the cell-level images imposes many challenges for building a practical HEp-2 CAD system. The most critical challenge is the requirement of cell segmentation step overhead. The HEp-2 individual cell segmentation is currently conducted using masks of the specimen images obtained through the DAPI binary mask [12]. DAPI is a fluorescent dye that delineates cells’ DNA regions to obtain ground truth masks. However, this added extra cost, effort, and time to the data acquisition process [13] in addition to the concerns about the carcinogenic nature of DAPI materials [14]. Furthermore, the severe skewness of the acquired cell-level HEp-2 mitotic-interphase data towards the interphase majority class further complicates the classification task, requiring additional data balancing approaches.

Motivated by the outstanding success of the recent deep learning object detectors in various imaging applications, and contrary to the previous works, this work proposes a deep learning-based multiple mitotic cell detection framework that could be implemented directly on the microscopic HEp-2 specimen images without segmentation and DAPI channel step overhead. The proposed mitotic cell detection framework is trained based on a deep active learning strategy to refine the detection ground truth (GT) labeling iteratively during training. The main contributions of this work are further highlighted as follows:

- An end-to-end deep learning framework is proposed to accurately and rapidly detect multiple mitotic cells from the entire HEp-2 specimen images within seven different classes of the I3A dataset: Centromere, Golgi, Homogeneous, Nuclear Membrane, Speckled, Nucleolar, and Mitotic Spindle. This is the first work that proposes detecting mitotic cells from the microscopic specimen image regardless of its class type, which provides a practical framework that could be integrated with a microscopic imaging system to support instant and direct detection of the mitotic cell from the captured specimen images.
- A deep active learning (DAL) strategy is involved to automatically annotate the mitotic cells for the detection task by adjusting the bounding boxes to surround each mitotic cell in the specimen images. Providing such annotation data could initiate further studies for developing mitotic cell detection approaches that could be applied to whole HEp-2 specimen images.
- A pre-processing step via contrast-limited adaptive histogram equalization (CLAHE) is employed to enhance the specimen image quality leading to improve detection performance.
- A comprehensive detection study is conducted by adopting two well-performed deep learning detectors (i.e., YOLO [15] and Faster R-CNN [16]) for detecting the HEp-2 mitotic cells to compare and select the best solution for this detection task.

The rest of this paper is organized as follows. A review of contemporary literature relevant to this study is presented in Section 2. Technical details of the proposed AI-based HEp-2 mitotic cell detection framework are provided in Section 3. The results and discussion of the experimental study are reported and discussed in Section 4. Finally, Section 5 summarizes the work findings and conclusion.

## 2. Related Works

Automatic detection of mitotic cells from various staining patterns of the entire HEp2 specimen images is challenging for building any CAD system for autoimmune disease [4,13,17]. The mitotic cell identification in the specimen images is a key to approving the precise slide preparation, indicating an important pattern similarity with specific interphase patterns that make the disease classification easier and present the rare unnoticed disorders during the microscopic inspection process by human experts [4]. The vast majority of previous works on HEp-2 image analysis have been proposed to solve the classification of interphase type HEp-2 staining patterns [4,13]. For instance, many initial studies attempted to approach this task using conventional learning methods, such as the works proposed in [12,18,19,20,21]. Alternatively, the later works proposed CNN-based approaches, which demonstrated superior performance over the conventional handcrafted-based learning methods [6,22,23,24,25,26,27,28].

### 2.1. HEp-2 Mitotic Cell Image Classification

**Figure 2:**
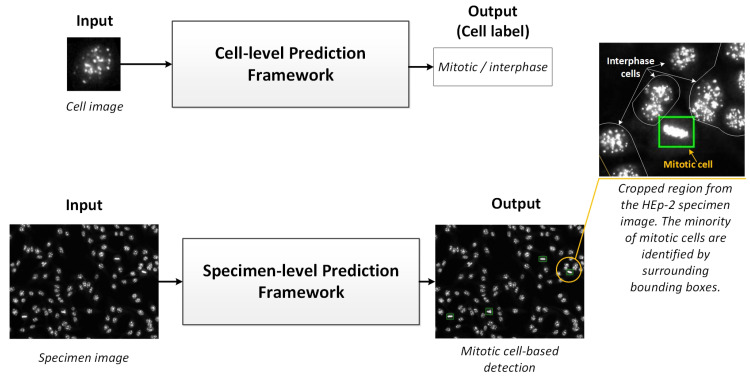
Mitotic cell prediction scenarios: Abstract view. The upper part shows the cell-level prediction framework, which requires cell image extraction overhead and then feeds them to the classification framework. In the lower pipeline, the prediction framework is designed to detect and classify mitotic cells from microscopic specimen images automatically and simultaneously.

Unlike the HEp-2 cell interphase patterns classification, a few research works have been conducted to solve the task of mitotic pattern detection from microscopic medical images for various diseases [10,13]. There are two kinds of deep learning approaches for predicting the mitotic cells: (1) HEp-2 single-cell image prediction, in which the individual cell images are segmented from the entire specimen image and processed to predict the class of each cell individually; and (2) HEp-2 specimen-image prediction, in which the entire specimen images are processed at once. For simplicity, the first and second approaches could be addressed as cell-level and specimen-level prediction scenarios, respectively. Figure 2 shows a schematic pictorial representation of both methods to process HEp-2 images. The cell-level prediction scenario is generally more accurate but needs exhausted user interventions regarding individual cell image extraction. Due to the availability of cell-level annotated datasets [13], cell-level-based approaches are commonly presented in the literature. Alternatively, the specimen-level scenario is preferable as a practical solution, especially with the scarcity of mitotic cell numbers in the HEp-2 samples. However, these methods have barely been studied in the literature due to the insufficiency of the annotated data [4]. The automatic detection of HEp-2 mitotic cells from the whole HEp-2 specimen images is important to develop reliable and feasible CAD systems to support the medical personnel in making rapid and accurate diagnosis decisions [4,13]. Moreover, improving the throughput capacity of the medical facilities reduces the variability among personnel/laboratories and minimizes the financial cost for such tests. In this section, we briefly describe the existing works for both cell-level as well as specimen-level prediction scenarios.

#### 2.1.1. Cell-Level Prediction Scenario

Almost all existing works in the literature on HEp-2 mitotic cell detection attempted to address this task as a binary classification between mitotic and interphase cell-level images. Because the mitotic cells are a minority in all available datasets, most existing works proposed using conventional learning approaches for solving the unbalancing problem. For instance, Foggia et al. [9] manually collected a small balanced dataset comprising 126 images from both classes (i.e., mitotic and interphase). They proposed using textural and morphological descriptors with an AdaBoost classifier for this task, achieving a maximum classification accuracy of 86.5% in their dataset. Using a different imbalanced dataset, Percannella et al. [29] proposed a threshold selection technique called multi-objective optimization for selecting the best classification decision among different classifiers and using different training scenarios. Their classifiers were trained on a heterogeneous set of morphological, texture, and LBP features [30]. However, their performance evaluation demonstrated poor results. In another work, Iannello et al. [10] proposed using a hybrid multi-expert system (MES) and multi-objective optimization method by training several classifiers with different data balancing scenarios. However, their method showed a poor result for classifying the positive mitotic class, achieving a true positive rate of 51.7%. Alternatively, Tonti et al. [31] suggested a completely unsupervised approach to differentiate the minority mitotic cell images from the remaining interphase cell images based on predefined rules on the morphological and texture GLCM [32] descriptors. The best classification accuracy from their method was achieved at 75.6%. Attempting to cope with data skewness, Miros et al. [11] proposed using both foreground fluorescent cell images with corresponding mask images to extract more representative features. Their classification algorithm was applied to a collection of shape, intensity, and texture features derived from the mask images.

Recently, this task was studied using various deep learning-based approaches. In [33], Gupta et al. extracted deep features from the different intermediate convolutional layers of the AlexNet. They pre-trained this network using the ImageNet dataset [34] and used the SVM for classification purposes. Afterward, Gupta et al. [35] proposed using a one-class SVM classifier to detect the nominal mitotic patterns among the majority of interphase patterns. In [36], Gupta et al. suggested using a Siamese deep network framework with a triplet loss function to address the unbalancing dataset by learning distance-based features. The SVM algorithm was used on the embedding-based features for classification. In [37], Gupta et al. recently proposed using a modified DCGAN generative model to generate new mitotic cell images for oversampling the minority class. Then, they extracted CNN-based and LM filter bank features from a mixture of real and manually selected GAN-synthesized mitotic images. For classification, they used the SVM algorithm. However, as shown by the evaluation results, the impact of adding the GAN-synthesized mitotic images was not clearly effective in improving the classification performance. In contemporary work, Anaam et al. [38] proposed an end-to-end deep learning model based on 1D-DCGAN on the feature space. In their work, a 1D-DCGAN model was trained to learn the feature distribution of the mitotic cells in the embedded space obtained by a CNN network. Then, the trained 1D-DCGAN generator was integrated within a CNN network during the training process to augment the mitotic sample features.

#### 2.1.2. Specimen-Level Prediction Scenario

Unlike the cell-level scenario, the specimen-level scenario performs the prediction task over the entire HEp-2 specimen images [13]. A few works in the literature were introduced for approaching different tasks of the HEp-2 CADs pipeline using specimen-level images. For instance, Oraibi et al. [39] proposed a hybrid method that employs the VGG-19 CNN model [40] and the handcrafted feature of LBP [30] and joint motif labels [41] to boost the discriminative capacity of CNN features. Cascio et al. [42] proposed a classification framework for the HEp-2 specimen images based on their individual cell images. Firstly, the cell images were extracted from the specimens using an active contours model algorithm (ACM). Then, a combination of intensity, shape, and texture features was used with the LDA selection method. Two-step classification was employed using class-aware binary SVMs and KNN. For specimen image segmentation and classification, Xie et al. [43] proposed a modified FCN [44] network that shares feature maps between the downsampling and upsampling parts of the network with a feature fusion strategy and additional skip connections. In a more recent work, Percannella et al. [45] proposed a U-Net-like model for jointly solving the tasks of specimen images intensity classification and segmentation on HEp-2 cell images in an end-to-end manner. A few works that proposed to solve segmentation and classification tasks of the interphase type of the HEp-2 specimen images [13].

However, to the best of our knowledge, the recent work presented by Gupta et al. [17] is the only existing work that partially addresses this task in specimen-level images. They proposed using a Faster R-CNN [16] algorithm to detect one type of mitotic cell called Mitotic Spindle to identify the Mitotic Spindle specimen images from the remaining interphase specimen classes. However, they ignore detecting other mitotic cells from the remaining different HEp-2 specimen classes [17]. Alternatively, for the first time, this work proposes an automatic deep learning-based framework for mitotic cell detection from all different classes of the HEp-2 specimen images: Centromere, Golgi, Homogeneous, Nuclear membrane, Speckled, Nucleolar, and Mitotic Spindle, which is essential to developing reliable and feasible HEp-2 CAD systems [4,13]. This work aims to contribute effectively toward developing such systems to support the medical staff in diagnosing rapidly and accurately, increasing laboratory throughput, and minimizing the cost of such tests.

### 2.2. GANs for HEp-2 Cell Image Analysis

Recently, increasing research has been conducted to study the use of GANs for different medical imaging applications involving detection tasks [46,47]. For example, Madani et al. [48] proposed a GAN-based semi-supervised learning approach to improve the detection performance of cardiac abnormality from X-ray imaging with less annotated data. Chen and Konukoglu [49] employed an adversarial auto-encoder model to detect lesion regions from brain MR images. Schlegl et al. [50] used an unsupervised fast AnoGAN (f-AnoGAN) to score the fitness of the unseen images to that of the normal images in the GAN-learned latent manifold to detect anomalies in the optical coherence tomography (OCT) images. For another imaging modality, Han et al. [51] proposed 3D Multi-Conditional GAN (MCGAN) to generate new volumetric nodules in lung CT images for augmentation purposes. Their MCGAN architecture was designed to conditionally optimize the generated nodules’ position, size, and attenuation to enhance the performance of a cascaded 3D CNN-based object detector. Nonetheless, various other contemporary research suggested using GANs to boost the classification performance of different medical imaging modalities, such as works in [52,53,54,55,56].

Alternatively, a limited number of studies that used GANs to approach the HEp-2 cell image analysis tasks are found in the literature. For example, Li et al. [57] used a U-Net generator with a modified conditional pix2pix [58] network to improve the HEp-2 cell segmentation performance. Kastaniotis et al. [59] proposed using Teacher-network to guide the attention maps in the DCGAN [60] discriminator network to improve the quality of the generated HEp-2 cell images. In another work, Xie et al. [61] used the pix2pix [58] configuration to synthesize cell-level mask images to improve the performance of a cascaded CNN classification model. Later, Majtner et al. [28] employed a separated DCGAN model to synthesize new images of each HEp-2 interphase type class for augmentation purposes. In more recent work, Anaam et al. [6] studied the effectiveness of using different GAN models to generate new HEp-2 cell images for boosting the CNN classification performances. However, for the specific task of HEp-2 mitotic cell classification, the works proposed by Gupta et al. [37] and Anaam et al. [38], introduced in Section 2.1.1, are the only published studies.

## 3. Materials and Methods

**Figure 3:**
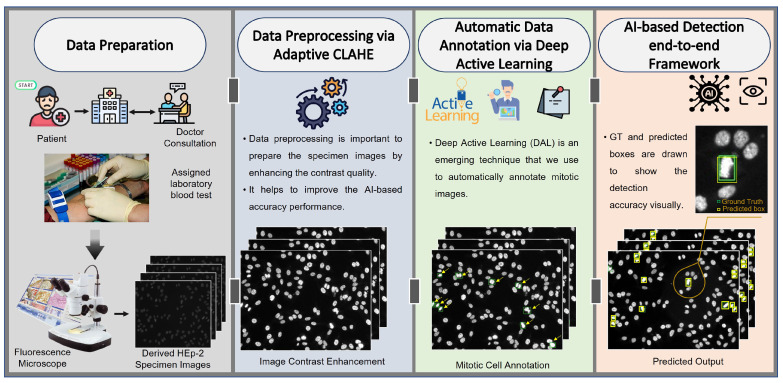
The proposed end-to-end AI-based framework for mitotic cell detection from the microscopic HEp-2 specimen images. The proposed framework involves four consecutive steps: data preparation, pre-processing, automatic labeling via deep active learning (DAL), and detection process via YOLO and Faster R-CNN.

The end-to-end scenario of the proposed AI-based mitotic cell detection framework is depicted in Figure 3. This framework has four sequential steps to achieve the final prediction goal of mitotic cells from the entire HEp-2 specimen images. The execution processing steps include medical HEp-2 data collection and preparation, data pre-processing, mitotic cell annotation, and the detection step based on the AI models. The technical concept of each processing step is explained in detail in the following sections.

### 3.1. Description of HEp-2 Medical Dataset

To achieve the goal of this work, the publicly available I3A Task-2 HEp-2 specimen dataset is used (Download link for the I3A dataset: https://hep2.unisa.it/dbtools.html (accessed on 20 March 2023)) [62]. This dataset was collected from 1001 cases with positive ANA samples at the Sullivan Nicolaides Pathology Laboratory in Australia. A monochrome camera mounted on a microscope with a Plan-Apochromat 20×/0.8 objective lens and Led illumination source was used for acquiring images with a resolution of 1388 × 1040 pixels from four distinct locations from each specimen slide. For each location, the imaging system acquires two different channel images: (1) the fluorescein-isothiocyanate (FITC) channel that carries the main contextual cell pattern information, which is typically used in the ANA tests; and (2) the 4’,6-diamidino-2-phenylindole (DAPI) channel, which is a fluorescent stain used to delineate the HEp-2 cell nuclei and generate ground truth masks for the HEp-2 specimen images. In total, this dataset contains 1,008 specimen images consisting of seven classes: the first six classes are identified based on the majority of the interphase type patterns, which are Centromere (Ce), Golgi (Gl), Homogeneous (Ho), Nucleolar (Nu), Nuclear Membrane (NuM), and Speckled (Sp). However, the seventh Mitotic Spindle (MS) class is associated with the minority mitotic phase patterns that exist in those slides. Each image is provided with its corresponding DAPI-stained ground truth mask image. Each specimen image was assumed to have a single HEp-2 pattern (i.e., interphase type or the Mitotic Spindle class), and hence was given a single label.

Each specimen slide (i.e., four patches’ images in the used dataset) should contain a few cells in the mitotic phase to validate the test preparation process. Thus, there is a need to manually annotate the mitotic cells across all specimen images to develop the proposed detection framework. Table 1 summarizes the data distribution over classes of the I3A Task-2 dataset. Figure 1 shows some examples of the HEp-2 specimen images with their corresponding DAPI binary masks.

**Figure 4:**
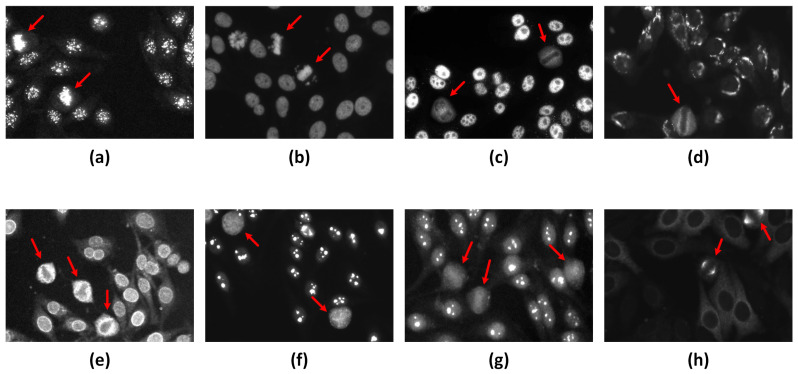
Examples of the different fluorescence patterns of the HEp-2 mitotic cells. The mitotic cells are annotated by red arrows. (**a**,**b**) Positive mitotic patterns that appear in Centromere and Homogeneous patterns, respectively. (**c**–**e**) Negative mitotic patterns appear in the Speckled, Golgi, and Nuclear membrane patterns, respectively. (**f**) Diffuse cytoplasmic mitotic patterns exhibited with Nucleolar pattern, while mitotic cells could appear with a positive pattern within the Nucleolar Specimen as shown in (**g**). (**h**) Mitotic Spindle pattern.

As mentioned earlier, HEp-2 cells undergo the mitotic phase in a small portion of their lifecycle, making their appearance in the specimen images very rare. Moreover, depending on the staining patterns of the specimen, the associated mitotic cells can manifest in different fluorescence patterns, as illustrated in Figure 4. For instance, for the centromere and homogeneous staining patterns, the mitotic cells exhibit positive mitotic patterns in which the mitotic cell body is weakly/non-fluorescent. In contrast, the chromosome mass located in the middle part of the cell shows denser fluorescence, as shown in Figure 4a,b. It is unlikely that the mitotic cells that appear in the speckled, golgi, and nuclear membrane patterns exhibit opposite negative mitotic patterns. The negative mitotic pattern is characterized by a fluorescent cell body (i.e., peripheral part) and non/weak fluorescent in the collapsed chromosome mass located in the central part of the cell, as shown in Figure 4c–e. Moreover, mitotic cells associated with the nucleolar pattern could appear as negative patterns with diffuse cytoplasmic staining in some cases, as shown in Figure 4f, or might exhibit positive staining of the chromosomal region in other cases, as depicted in Figure 4g [63]. However, the Mitotic Spindle pattern is characterized by the stained spindle fibers that form a cone-shaped decoration for the poles of the mitotic cells, as shown in Figure 4h.

### 3.2. Medical Data Preparation

**Figure 5:**
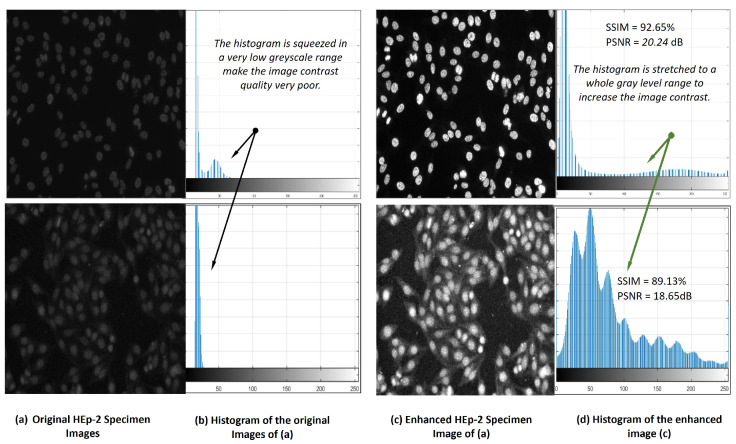
HEp-2 specimen image contrast quality improvement via contrast-limited adaptive histogram equalization (CLAHE) technique.

As it is known that the positive HEp-2 specimen images can be acquired with low contrast quality [12]. To improve the image contrast quality and help AI models for better prediction performance, contrast-limited adaptive histogram equalization (CLAHE) is applied [64,65]. Using CLAHE on the original HEp-2 specimen image, the histogram is stretched to cover the whole range of the intensity greyscale, enhancing the HEp-2 image contrast. Figure 5 shows an example of the pre-processing step based on the image processing histogram equalization technique. It is clearly shown that the image quality is enhanced by increasing the image contrast, and all cells become more distinguishable. Meanwhile, the structural similarity index (SSIM) indicates the quality of the image by improving its contrast with 92.65% and 89.13% for these two examples. As a part of the data pre-processing, the HEp-2 specimen images are normalized in the range of [0, 255] to improve the overall prediction performance of the AI-based models [65,66]. All images are resized using bi-cubic interpolation to scale their intensity pixels into the same range of 460 × 600 pixels.

### 3.3. Data Annotation via Deep Active Learning (DAL)

**Figure 6:**
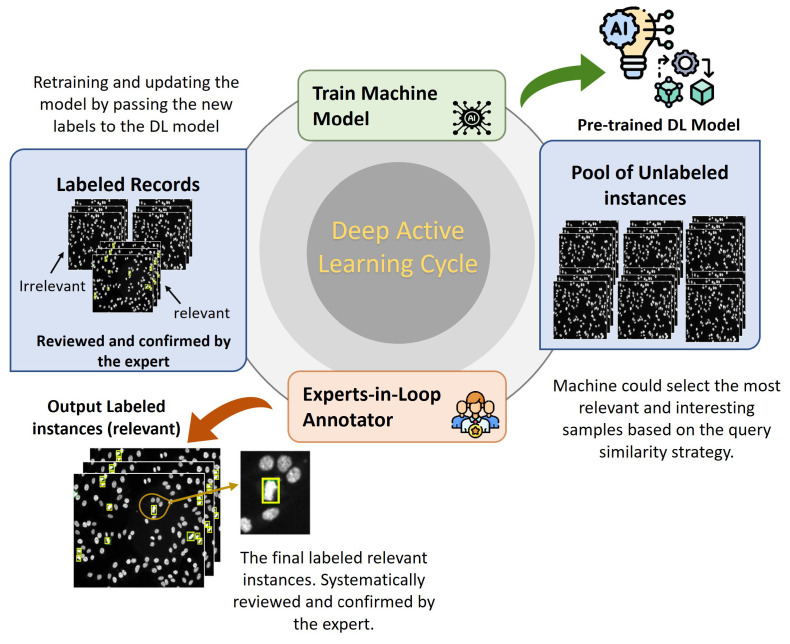
Deep active learning (DAL) strategy to automatically annotate the specimen HEp-2 images for the mitotic cell detection task.

Deep active learning (DAL) is an emerging approach that automatically annotates unlabeled data to fine-tune the supervised learning algorithms accurately [67,68]. The concept of DAL is depicted in Figure 6. Indeed, data annotation is a time-consuming and expensive process when dealing with a large amount of unlabeled data. To avoid having manual annotation for the entire dataset instances, DAL could iteratively label the data samples that are assumed to maximize the deep learning model quality while minimizing the annotation efforts and time. In general, the importance of the DAL approach for automatic data labeling can be summarized as follows:

- Efficient Use of Labeling Resources: Labeling large datasets can be time-consuming and expensive. DAL can significantly reduce the manually labeled data needed to achieve high performance in a deep learning model, saving significant labeling resources.
- Improved Model Performance: DAL can lead to more accurate deep learning models. By selecting the most informative samples to label, the model can learn from high-quality data and improve its performance faster than it would with random sampling.
- Active Learning: DAL incorporates active learning, which is an approach that seeks to optimize the learning process by selecting the most informative samples to label. This can lead to faster convergence of the model and better generalization performance.
- Human-in-the-Loop: DAL can also incorporate human feedback into the labeling process. This can further improve the performance of the model by taking advantage of human expertise and intuition.

To detect the mitotic cells among all other cells in the same HEp-2 specimen image, the machine should learn the different patterns of mitotic cells of all the seven classes based on the ground truth (GT) labels. For the object detection task, the GT is the bounding box that surrounds the whole target object (i.e., mitotic cell) inside the HEp-2 specimen image. To automatically generate the GT labels for all HEp-2 specimen images, the DAL procedure includes the following strategies: initialization strategy, deep learning model selection, query strategy, and stopping criterion. For the initialization strategy, a sub-set of HEp-2 specimen images from all seven classes is carefully selected and manually annotated with the support of the GT binary DAPI masks shown in Figure 1. The GT bounding boxes are generated to embrace each mitotic cell inside a specific HEp-2 specimen image.

Three experts are involved in verifying the mitotic cell appearance in the specimen images to generate trustable GT labels. Two state-of-the-art deep learning detectors (i.e., YOLO and Faster R-CNN) are selected for the model selection as they are well-known and reliably used for detection tasks in the medical imaging domain [64,65,69,70]. Both detectors are pre-trained using the initial sub-set of annotated HEp-2 specimen images. Both detectors are evaluated during the iterative active learning process, and the best model is selected. Once the deep learning detector is pre-trained, the remaining unlabeled specimen images are iteratively used to predict the potential mitotic cells. For the query strategy, the similarity between the predicted data in the first round of DAL and the initial subset is measured. Then, the specimen images with the highest similarity are selected by the machine, called the relevant or interesting sample images. These images are passed to the domain experts-in-loop to check, modify, and confirm the automated labeling process. They are able to manually adjust the generated bounding boxes’ location, add missing bounding boxes for the unseeing objects, or even delete the wrong detected bounding boxes. For the stopping criterion, once all HEp-2 specimen images are correctly annotated, the DAL cycle is terminated. After dataset preparation and labeling steps, the dataset is randomly divided per class into 80% for training and 20% for testing. The validation set is represented by 10% of the training set. Since each patient has four different HEp-2 slices, we carefully group and include them in only one training, testing, or validation set to avoid any biases of the network’s weights fine-tuning during the training time.

### 3.4. Data Augmentation

**Figure 7:**
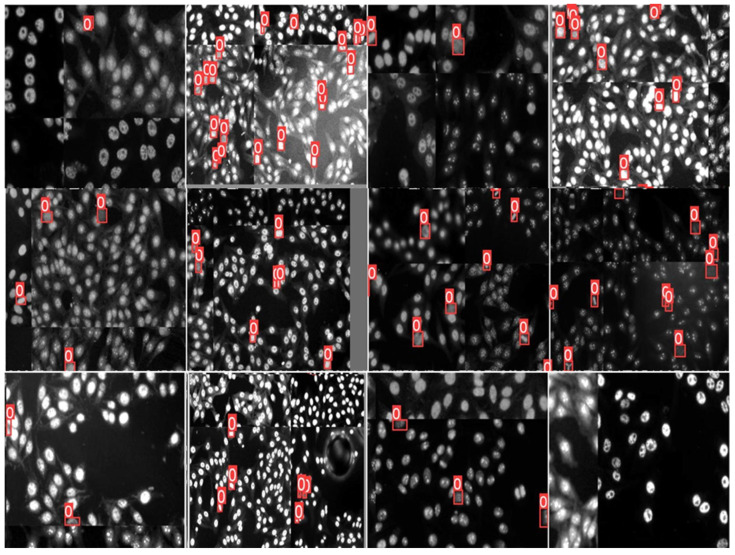
Examples of specimen images obtained by the used data augmentation techniques.

We enlarged the size of the training dataset via various augmentation strategies to improve the prediction accuracy, avoid the over-fitting that may occur during the training time, and also to minimize the class imbalance problem [65]. We apply image photometric and geometric distortions, which are recent augmentation strategies used to increase the number of training instances [71]. For photometric distortion, the hue, saturation, and value of the HEp-2 specimen images are adjusted by 0.015, 0.7, and 0.4, respectively. To perform the geometric distortion, random scaling of 0.9, translation of 0.1, and rotation lift-right of 0.5 are applied for training HEp-2 specimen images. In addition, Mosaic and MixUp augmentation methods are used with probabilities of 1 and 0.1, respectively. Figure 7 shows some augmented training HEp-2 specimen images.

### 3.5. The Concept of the AI-Based Mitotic Cell Detection

The detection of mitotic cells from the microscopic HEp-2 specimen images is achieved using robust deep learning detectors of YOLO [15] (i.e., YOLOv5) and Faster R-CNN [16]. These detectors are selected due to the following reasons. First, they have a high capability to detect multiple objects (i.e., mitotic cells) accurately and rapidly [65,70]. Second, simultaneously, they can estimate the confidence scores using the last neural network layer for each detected bounding box [72]. Third, these methods can detect objects with different sizes (predicts different bounding boxes’ sizes), as they are equipped with a learnable bounding box coordinates regression function. Fourth, YOLO is known for its fast inference time, making it a good choice for real-time applications where speed is critical. Faster R-CNN also has a fast inference time compared to other R-CNN models. Fifth, YOLO, and Faster R-CNN have shown high accuracy in object detection tasks, with YOLO achieving higher mean average precision (mAP) on some datasets. YOLO and Faster R-CNN are known for their ability to detect objects at different scales and aspect ratios, making them good choices for detecting objects in various environments. Both algorithms have well-established medical imaging implementations, demonstrating high performance in the detection tasks.

#### 3.5.1. Mitotic Cell Detection Based on YOLO Predictor

**Figure 8:**
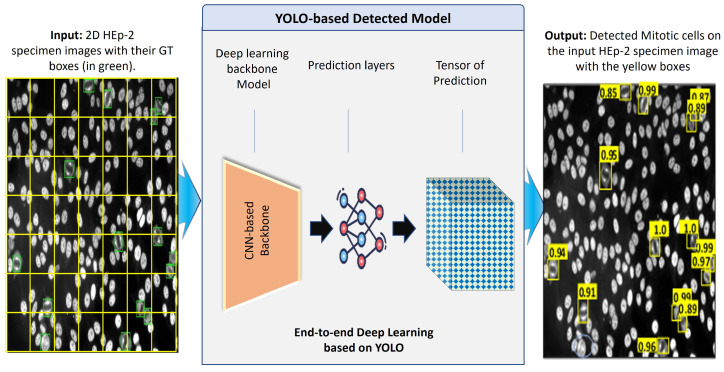
Deep learning YOLO detector for mitotic cell detection from the microscopic HEp-2 specimen images.

Figure 8 shows the abstract view of the deep learning components of the YOLO predictor [15]. YOLO has a deep learning structure designed to directly detect the objects (i.e., mitotic cells in our case) from the input microscopic HEp-2 specimen images without user interventions [64,65,70]. Accurate and rapid object detection plays a crucial role in real-time AI applications. Thus, the one-stage detector based on the YOLO-v5 is selected to perform the mitotic cell detection for this research study. Generally, deep learning detectors involve two main stages [64,65,70]. The first stage is composed of a CNN-based backbone network to generate deep high-level features from the input image. The head predictor detects the bounding box coordinates surrounding the mitotic cells in the second stage. Recently, the YOLO deep learning structure was modified by inserting additional deep layers between the backbone and the head network to formulate a third part called the neck network [71], which plays a vital role in extracting millions of different features. This is the key behind the deep learning models to learn a huge number of features about the target objects and then detect them accurately.

Several deep learning networks have been developed recently in the literature, such as VGG, ResNet, DenseNet, Swin Transformer, and CSP with SPP. Once the backbone network derives the deep features, the neck network interacts and plays its role between the backbone and heads to utilize the deep features effectively. The neck network has multiple bottom-up and top-down deep learning paths designed by different deep learning convolutional layers for reprocessing and rationally using the extracted features from the backbone network. Finally, the head layers are used to fine-tune and predict the bounding box coordinates of the potential objects in the input HEp-2 specimen image. In such cases, the output predicted boxes have several values, such as the bounding box coordinates: center (x,y), width (w), and height (h). Concurrently, the confidence score representing the probability of object existence is estimated for each box using the neural network layers. For each prediction output, a specific node in the network is responsible for optimizing and finding the best solution. At the end, all predictors are stored in the tensor of prediction, as shown in Figure 8.

#### 3.5.2. Mitotic Cell Detection Based on Faster R-CNN

Faster Region-CNN (Faster-RCNN) [16] is an efficient deep CNN-based framework demonstrating excellent performance in object detection tasks. Recently, it has been widely applied for abnormality detection in medical imaging. For this work, the Faster R-CNN is adopted and used as a holistic framework for detecting mitotic cells from the entire HEp-2 specimen images. This algorithm takes the whole HEp-2 specimen images as an input and predicts the locations of mitotic cells in terms of bounding box coordinates. Simultaneously, it could predict the detection probability for each detected bounding box, indicating the object’s existence probability.

Faster R-CNN deep architecture is composed of a backbone pre-trained network (CNN), region proposal network (RPN), region of interest (RoI) pooling, and head network. First, the backbone pre-trained CNN extracts the most representative deep feature maps from the input HEp-2 specimen images. This network takes the input images and yields their corresponding feature maps. Different CNN architecture could be employed as a feature generation component of the Faster R-CNN. In this work, we use a backbone of the ResNet-50 network [73], which is pre-trained on the large ImageNet dataset [34]. Then, the model is fine-tuned using our labeled dataset during the training process. Second, the region proposal network (RPN) is a simple network composed of two FC layers that take the feature maps extracted by the backbone network as input and generates class-agnostic region proposals (i.e., bounding boxes). In particular, RPN is trained to associate reference pre-defined fixed-sizes boxes placed uniformly throughout the image (known as anchors) with the truth bounding boxes by optimizing the regression error between them. The output of this network is a list of proposed bounding boxes’ coordinates along with their probabilities of occurrence. In our implementation, we modified the RPN anchor boxes to be suitable to effectively cover the size range of the mitotic HEp-2 cells with the sizes of $32^{2}$, $64^{2}$, and $128^{2}$ pixels. At the same time, the default settings of aspect ratios are kept intact as 1:1, 1:2, and 2:1, and the overlapping threshold of 0.7 is used. The RPN output is refined using a non-maximum suppression (NMS) algorithm to eliminate the duplicated or unwanted bounding boxes.

**Figure 9:**
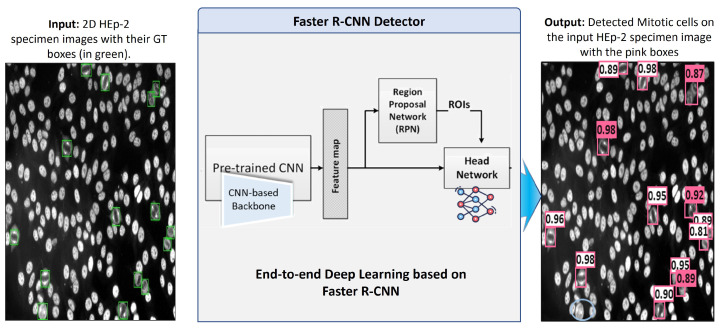
Deep learning Faster R-CNN detector for mitotic cell detection from the microscopic HEp-2 specimen images.

The region of interest (RoI) pooling layer uses the feature maps generated by the backbone network to extract features relevant to each proposed bounding box and resize them to a fixed size (i.e., 7 × 7 × C, where C is the channel dimension of the feature map). Finally, the head network is a simple two fully connected layers used for final object classification and refinement of bounding boxes’ coordinates. Deep learning components of the Faster R-CNN are trained using the labeled specimen images that provide the bounding boxes’ coordinates for each mitotic cell inside the microscopic HEp-2 image. The abstract diagram of the deep learning Faster R-CNN architecture is depicted in Figure 9.

### 3.6. Experimental Setting

A multi-scale strategy is used during training to learn the mitotic cell prediction via multiple image spatial resolutions [74,75]. The hyper-parameters are experimentally optimized and selected to achieve the best mitotic cell detection accuracy. Whereas, a trial and error strategy is used to experimentally optimize and choose the training settings for both predictors separately [65,70].

For the YOLO detector, a mini-batch size of 32 with 100 epochs is selected to fine-tune the AI models. For optimization, the stochastic gradient descent (SGD) optimizer is selected with the initial learning rate (LR) of 0.01, the final one-cycle learning rate of 0.1, the momentum of 0.937, weight decay of $5\times 10^{-4}$, warmup epochs of 3, warmup momentum of 0.8, and warmup initial bias learning rate of 0.1. For the Faster R-CNN detector, the implemented Faster RCNN model was trained using the SGD optimizer with an initial learning rate of 0.001 and a decay factor of 0.1 every 25 epochs. The model is trained for 100 epochs using the momentum of 0.9, weight decay of 0.0005, and batch size of 1. For both models, the gains of box loss, class loss, and object loss are adjusted to be 0.05, 0.3, and 0.7, respectively. The training IoU threshold and anchor-multiple threshold are selected as 0.2 and 4, respectively.

### 3.7. Performance Evaluation Strategy

The proposed deep learning detection framework is evaluated using a 5-fold cross-validation strategy using the testing sets. For each fold, the quantitative evaluation results of precision, recall, and mean average precession (mAP@0.5) are derived and reported, which are defined as follows:

- Precision: measures the ratio of the predicted true positives (TP) to all positive predictions (TP + FP):
  (1) $$Precision=\frac{TP}{TP+FP}$$
- Recall: measures the ratio of the predicted true positive (TP) to the total positive (TP + FN):
  (2) $$Recall=\frac{TP}{TP+FN}$$
- Mean Average Precision (mAP): measures the performance of object detection models by calculating the mean of average precision (AP) values over different recall values at a specific IoU threshold:
  (3) $$mAP=\frac{1}{N}\sum_{i=1}^{N}AP_{i},$$
  where N is the number of classes, and $AP_{i}$ is the average precision of class *i*. AP represents a weighted average of precision at different recall values, and is calculated as follows:
  (4) $$AP=\sum_{k=0}^{n-1}\left[Recall\left(k\right)-Recall\left(k+1\right)\right]\times Precision\left(k\right),$$
  where n is the number of thresholds, recalls(n)=0, and precisions(n)=1. During the calculations, the detection is considered TP if the IoU between the predicted and the GT bounding box is greater than the pre-specified threshold (i.e., 0.5 in our case).

Each evaluation metric is monitored during the training process to show the detection improvement over the training time. In addition, the training and validation bounding boxes regression and detection loss curves are also reported. Meanwhile, some qualitative results of the predicted mitotic cells on the entire HEp-2 specimen images are visually presented to show the potential predicted bounding boxes with their confidence probability scores.

### 3.8. Execution Environment

The experimental study is executed via a PC with a CPU of Intel(R) Core(TM) i7-10700KF @ 3.80 GHz, 32.0 GB RAM, six CPUs, and one GPU of NVIDIA GeForce RTX 3060.

## 4. Results and Discussion

### 4.1. Training/Validation Performance

**Figure 10:**
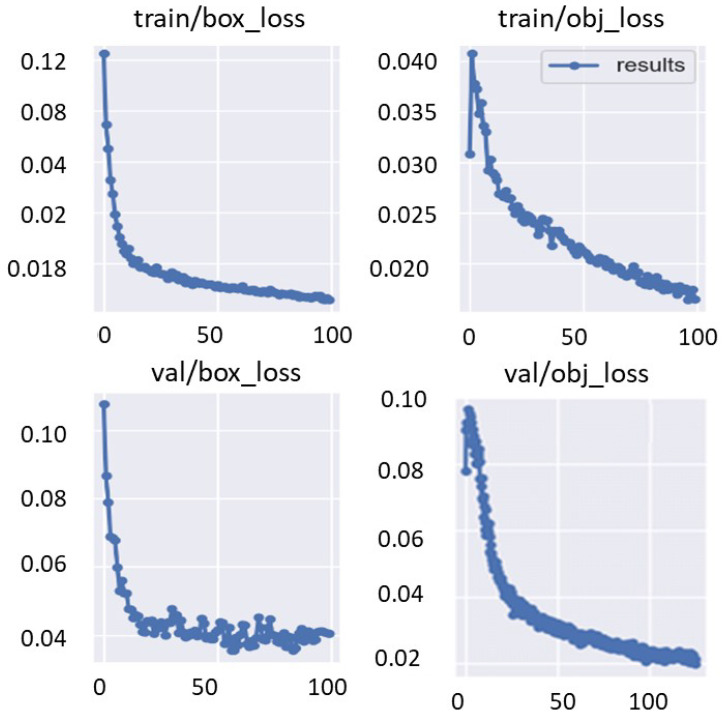
Training parameter optimization of train/valid bounding boxes regression and object detection losses functions. These curves are recorded using Fold-3 training/validation sets with YOLO detector.

**Figure 11:**
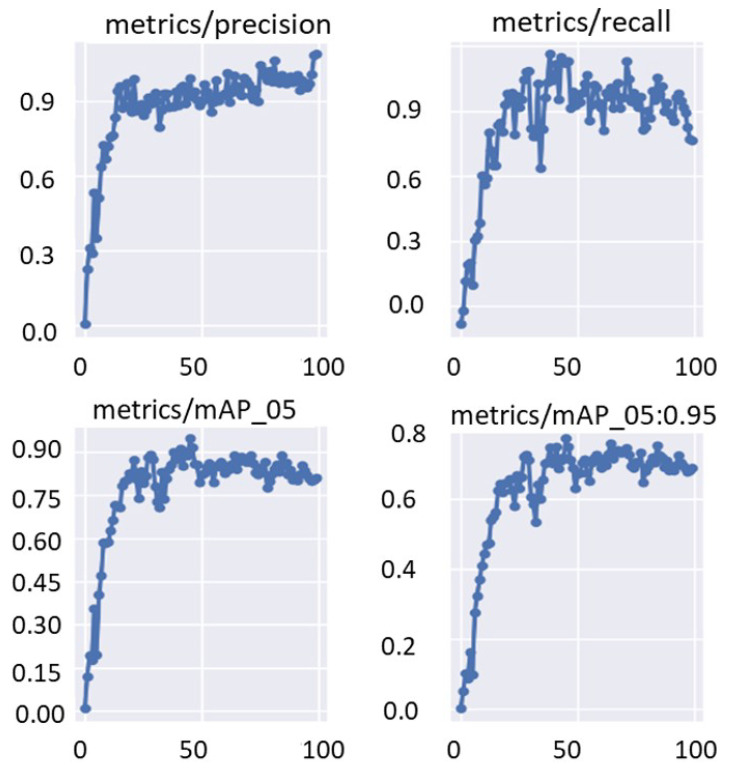
Validation performance in terms of precision, recall, and mAP. These curves are recorded using Fold-3 training/validation sets with the YOLO detector.

The training and validation bounding box regression and object detection loss functions are recorded for each epoch during the fine-tuning optimization process of the deep learning models. This is to evaluate the deep learning parameter optimization progress during the training process. Figure 10 shows an example of the train/valid bounding box and object loss function curves over 100 epochs. Moreover, Figure 11 shows the evaluation metrics of precision, recall, and mAP that were recorded for the same training settings used to record the loss function curves of the validation set. It is clearly shown that the evaluation metrics improved with increasing the training epoch. This means that the deep learning detectors are learned well without any overfitting to the training data.

### 4.2. Evaluation of Data Labelling via DAL

**Figure 12:**
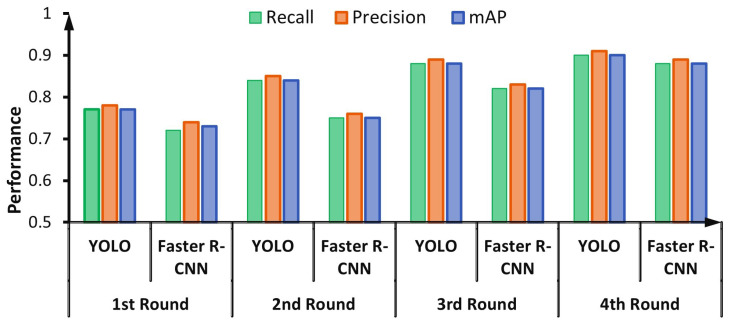
Iterative cycle detection performance for the deep active learning (DAL) during the mitotic cell automatic annotation process.

For labeling HEp-2 mitotic cell bounding boxes, the DAL cycle is iteratively repeated four times. At each cycle, the evaluation of both detectors is checked and recorded. Figure 12 shows the capability of detectors to learn better in each round of the DAL cycle. The performance of both detectors is increased with each cycle because the number of annotated images is increased iteratively, as explained in Figure 6.

For the first DAL cycle, the subset of 200 HEp-2 specimen images is manually annotated by three experts as mentioned in Section 3.3. Both models of deep learning detectors are pre-trained using the selected 200 subset images at the first cycle. Using the rest of the unannotated images, both detectors are evaluated. After that, the highest similarity images with the first subset are passed and verified by the expert in the loop to check and modify the machine prediction and send them again for the second DAL cycle. This process is repeated four times until all HEp-2 specimen training images are totally annotated.

For the YOLO detector, the validation performance in terms of precision, recall, and mAP increased from 77.01%, 78.56%, and 72.98% in the first round into 90.26%, 89.53%, and 86.35% in the fourth round, respectively. Similarly, the validation performance of the Faster R-CNN during the DAL rounds increased from 70.15%, 69.89%, and 65.89% in the first round into 88.85%, 87.98%, and 85.61% in the fourth round in terms of precision, recall, and mAP, respectively. It is clearly shown that the YOLO detector could achieve a better detection evaluation over all DAL training cycles, as shown in Figure 12.

**Figure 13:**
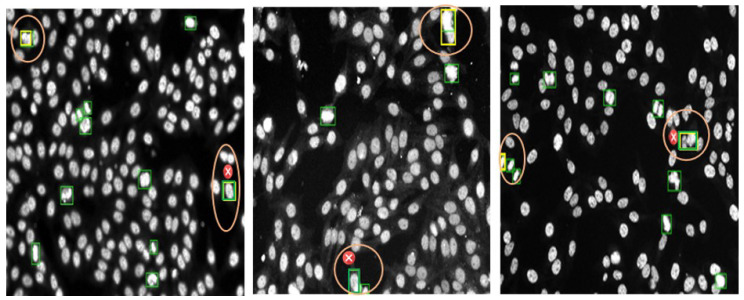
Examples of mitotic cell annotation process during the third DAL cycle. This is to show the Expert-in-Loop interaction with the machine to verify, modify, and confirm the labeling procedure. The left and middle images show the annotation using YOLO, while the right one is annotated via Faster R-CNN. The automated mitotic cell labels are depicted in the green boxes, while the adjusted labels by the expert-in-loop are superimposed by yellow boxes surrounded by orange circles. The falsely detected boxes (i.e., removed boxes) are annotated by small red circles with “x” signs.

Figure 13 shows examples of the annotated HEp-2 specimen images for mitotic cells via the DAL strategy at the third cycle. The green boxes represent the machine prediction of the mitotic cells in each specimen image. In contrast, the expert-in-loop interactions with the machine to modify, remove, or add bounding boxes annotation for the mitotic cells are highlighted with yellow boxes inside circles in the figure. The experts in the loop remove the false positive (FP) cases that appear with cross signs in Figure 13. Furthermore, the expert added the missing boxes of the false negative (FN) cases that were missed by the machine but are TP. Such a case appears in the circle at the top of the middle image in Figure 13. Moreover, the sizes of the detected boxes are adjusted to involve the whole object body, as shown in the circles in the left and right images of Figure 13. It is clearly shown that multiple mitotic cells from different classes could be effectively annotated via a machine with experts during the DAL cycles.

### 4.3. Detection Results

The quantitative detection evaluation results using YOLO and Faster R-CNN detectors are summarized in Table 2. The mitotic cell detection results are recorded for each testing fold via the deep learning models trained using the same deep structure and training settings. For the YOLO detector, the average evaluation results in terms of recall, precision, and mAP are achieved with 90.01%, 88.30%, and 81.53%, respectively. For Faster R-CNN, the average detection performance for recall, precision, and mAP are 86.98%, 85.28%, and 78.50%, respectively. Indeed, both detectors could extract the mitotic cells from the different classes of the HEp-2 specimens: Centromere, Golgi, Homogeneous, Nuclear Membrane, Speckled, Nucleolar, and Mitotic Spindle. Using the YOLO algorithm for mitotic cell detection demonstrated relatively better results. As noted from Table 2, YOLO could correctly predict mitotic cells with average TP cases more than Faster R-CNN. Such results encourage adopting the YOLO-based framework as a practical solution for supporting the detection of mitotic cells from the microscopic HEp-2 specimen images regardless of their interphase staining type.

Moreover, a paired *t*-test is used to statistically examine the significance of the differences between the results of both detectors. The null hypothesis (Ho) is that there are no significant differences between the results of both detectors, while the alternative hypothesis (Ha) is that their results are significantly different. Using a confidence level of α=0.05, *t*-test analysis demonstrated significant differences between the effects of YOLO and Faster R-CNN with p-values of $1.1467\times 10^{-16}$, $4.6848\times 10^{-60}$, and $1.0523\times 10^{-59}$ for the comparison between results of recall, precision, and mAP, respectively. As a result, based on the *t*-test analysis, the YOLO detector performed better than the Faster R-CNN on HEp-2 mitotic cell detection from the microscopic specimen images.

**Figure 14:**
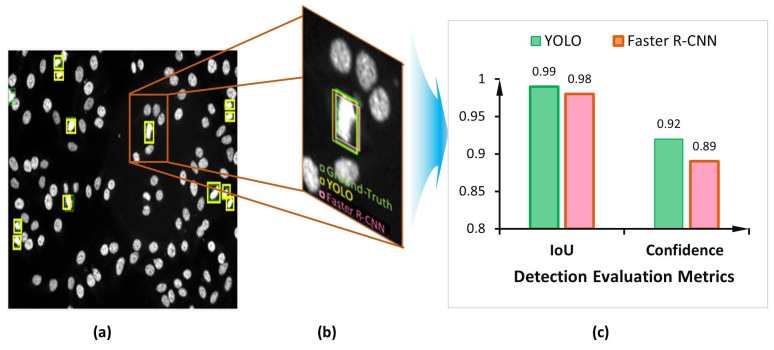
Example of the YOLO and Faster R-CNN qualitative detection results for the task of mitotic cell detection from the microscopic HEp-2 specimen images: (**a**) The output HEp-2 specimen image with the predicted mitotic cells; (**b**) Zoomed in a small region from the HEp-2 specimen image that contains a single mitotic cell; (**c**) Detection score comparison of both the YOLO and Faster R-CNN detectors.

**Figure 15:**
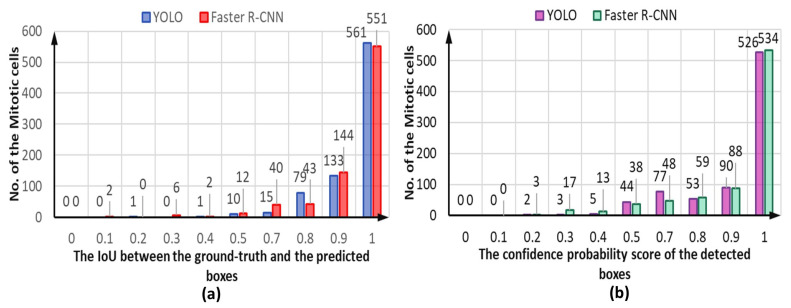
The capability of both YOLO and Faster R-CNN for accurate detection of the mitotic cells in terms of the (**a**) intersection over union (IoU) and (**b**) confidence probability threshold of the detected objects. These results are recorded from the third-fold trial.

Both detectors demonstrate accurate cell object detection and bounding box coordinate prediction for the mitotic cells, as shown in the example depicted in Figure 14. The evaluation process shows a high overlap between the ground truth boxes and their corresponding predicted boxes, achieving an IoU score of larger than 0.9 for most of the detected mitotic cells for both YOLO and Faster R-CNN detectors, as shown in Figure 15a. However, the YOLO model could predict slightly more accurate boxes regarding the IoU counting 561 optimal detected cases compared to 551 for the Faster R-CNN. In contrast, the number of correctly detected mitotic cells at IoU = 90% is calculated to be 133 and 144 cases for YOLO and Faster R-CNN, respectively. In less than 50% of IoU, the Faster R-CNN predicts 10 cases, while YOLO detects only 2. Such cases are considered to be weakly detected since the IoU is very low. Meanwhile, the higher confidence score proves that deep learning models could predict the mitotic cells correctly with high objectness probability. Figure 15b shows the capability of both detectors to predict the mitotic cells with a high confidence score for most of the testing HEp-2 specimen images.

**Figure 16:**
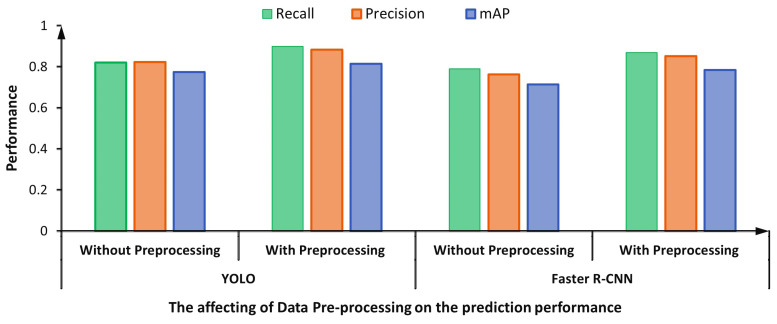
Effect of pre-processing on the mitotic cell detection performance: YOLO Vs. Faster R-CNN.

The pre-processed step for the HEp-2 specimen images using the CLAHE technique for contrast stretching improves the detection performance [65]. Specifically, such a pre-processing step supports the deep learning YOLO and Faster R-CNN detectors to achieve better evaluation results, as shown in Figure 16. The enhanced HEp-2 images gain improvements on their histogram in terms of the sharpness of the adjacent pixels to be more distinguishable, which improves the images’ spatial contrast quality making the detection process easier. This might justify the improvement in the prediction performance using the CLAHE technique. For the YOLO detector, the detection evaluation results are improved by 0.08, 0.06, and 0.04 in terms of recall, precision, and mAP, respectively. Similarly, the detection results of the Faster R-CNN are improved by 0.08, 0.09, and 0.07 in terms of recall, precision, and mAP, respectively.

**Figure 17:**
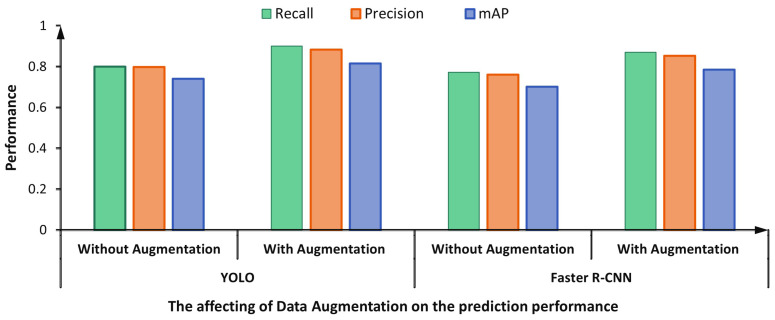
The prediction performance of the proposed framework via YOLO Vs. Faster R-CNN using the original and augmented datasets.

Figure 17 shows the impact of the adopted data augmentation techniques on the overall prediction performance of the proposed framework. The figure compares the performance of two dataset versions: original (i.e., without augmentation) and augmented datasets. It is clearly noticeable that both detectors achieved better detection performance by augmenting the training dataset. For instance, the recall, precision, and mAP are improved by 0.10, 0.085, and 0.075, respectively, for the YOLO detector. Similarly, the Faster R-CNN yielded higher scores by 0.098, 0.092, and 0.083 regarding recall, precision, and mAP, respectively.

**Figure 18:**
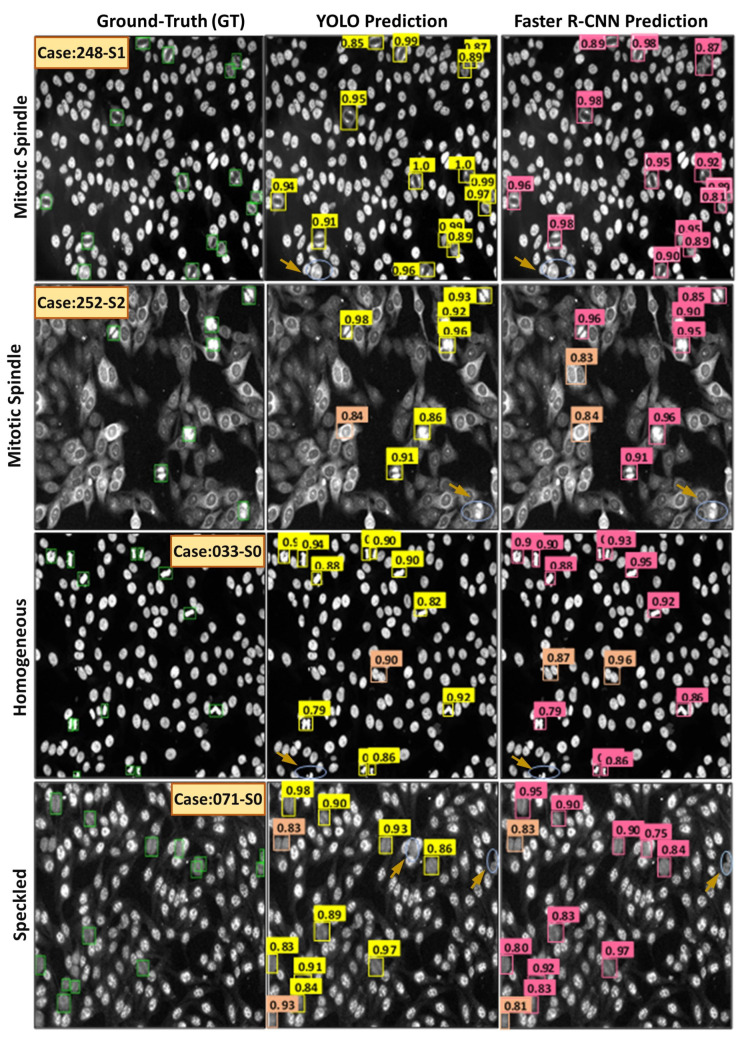
Some qualitative examples of detecting mitotic cells from the entire HEp-2 specimen images via YOLO and Faster R-CNN. The first column shows the ground truth bounding boxes in green. The true positive (TP) detected cases are labeled by yellow and pink boxes for YOLO and Faster R-CNN predictors, respectively. The false positive (FP) cases are highlighted by coral boxes for both detectors, while the false negative (FN) cases are highlighted by blue circles indicated with brown arrows.

Figure 18 shows examples of the HEp-2 mitotic cell detection results acquired using both YOLO and Faster R-CNN detectors. Both models could accurately detect most mitotic cells from different HEp-2 specimen images regardless of the class type. True positive (TP), false positive (FP), and false negative (FN) cases are annotated with different color circles, as shown in Figure 18. The TP cases represent the number of mitotic cells that are detected as mitotic. In contrast, the number of non-mitotic cells predicted as mitotic cells are the FP cases, while the FN cases mean the number of true mitotic cells the model does not detect. The falsely detected cases might be raised because multiple mitotic cells from different classes are involved in this study. The variability in the HEp-2 mitotic cell appearance is the top reason for detection failure. Whereas, HEp-2 mitotic cells manifest in different morphologies depending on the stage of the cell division process. Specifically, some mitotic cells show high morphological and textural similarity with other interphase patterns, which makes it challenging for the detection algorithm to identify all mitotic cells accurately.

The recent work of Gupta et al. [17] detects only the spindle mitotic cells ignoring the mitotic cells from other classes such as centromere, golgi, homogeneous, nuclear membrane, speckled, and nucleolar, which limits their prediction methodology to be applied as a practical solution. Alternatively, our proposed methodology aims to detect the mitotic cells from all HEp-2 staining classes. Such a comprehensive CAD system framework could be applicable in practical applications to minimize user intervention and labor concentration.

The execution time comparison for a single image detection is reported in Table 3. The YOLO detector needs 600 s to train per epoch, while the faster R-CNN requires around 1225 s. This is due to the deep learning structures of both detectors and the separated RPN network of the Faster RCNN model that has different capabilities to reach their training process in an epoch. For testing a single HEp-2 specimen image with the same number of mitotic cells, the YOLO algorithm outperforms the Faster R-CNN by 0.545 ms. The rapid detection strategy for YOLO makes it preferable for practical real-time applications. Meanwhile, YOLO could predict 5.56 frames per second, while Faster R-CNN predicts 3.03 specimen images per second regardless of the number of cells inside a single slide.

#### 4.3.1. Comparison with Existing Works

Research studies for HEp-2 mitotic cell detection are limited in the literature. To the best of our knowledge, this is the first deep learning study to detect multiple mitotic cells directly from the microscopic HEp-2 specimen images of different HEp-2 interphase-type staining classes. As mentioned above, Gupta et al. [17] used the Faster R-CNN detector to only identify spindle mitotic cell types for classifying the Mitotic Spindle specimen images from other specimen image classes. However, they ignored the mitotic cells existing in the other specimen classes, making the comparison with their results unfair. Their model was trained to learn deep features from only the spindle mitotic cells, making their model biased toward specific features and ignoring other types of mitotic cells from the remaining classes.

On the other hand, all previous works that addressed the mitotic-interphase classification task were proposed on cell-level images. In particular, a small subset of carefully cropped mitotic cells was classified against another set of interphase cell images as provided in the I3A Task-3 dataset [62]. Accordingly, the results achieved by those methods are relatively higher as the binary classification problem is simple and straightforward. However, those methods require careful segmentation for all HEp-2 cell objects beforehand, which is not practically suitable. Therefore, the direct comparison between the results of our mitotic cell detection framework with those cell-level classification approaches is not fairly applicable. However, Table 4 provides a comparison summary between the proposed detection framework against other recent existing works.

#### 4.3.2. Limitations and Future Work

The limitations of this work are associated with the scarcity of medical annotated datasets for models’ training, which imposes the use of the DAL mechanism for automatic data annotation. As known, the DAL procedure requires intensive labor work, especially from the experts who are responsible for verifying and confirming the automatic labels of the machine. The similarity among the mitotic cells with some interphase cells confuses the deep learning detectors in some sense predicting FP and FN cases. Even so, we experimentally optimized the training parameters of both detectors to achieve the best detection performance and minimize the falsely detected cases. It is required to improve this framework to make detection for all cell types to develop a comprehensive one-shot HEp-2 cell detection framework, which is challenging due to the lack of an accurate annotation for all individual cell objects in each specimen image.

A prospective future extension of this work is to extend the annotation task to include the interphase cell patterns of the HEp-2 specimen images to develop an end-to-end trainable framework for simultaneously classifying mitotic and interphase cell types directly from the microscopic HEp-2 specimen images. In contrast to recent related works such as that of Xie et al. [61], which assign a single class label for each specimen image and cannot identify the mitotic cells among the interphase type slides, the prospective framework is proposed to instantly detect and classify all cell objects in the specimen image individually. Accordingly, specimen images with mixed patterns could be properly diagnosed. To achieve this goal, an intensive effort is required from field experts in the process of cell annotations and qualitative performance evaluation.

Inspired by the recent success of using GANs for medical data augmentation [56], a potential solution to mitigate the problem of annotated data scarcity is by optimizing conditional variants of GANs (e.g., pix2pix [58] or CycleGAN [80]) for augmenting the HEp-2 medical data [51]. Moreover, more recent detection algorithms such as EfficientDet [81] are planned to be implemented for this task in order to achieve the highest detection performance. Using the entire specimen image, all ANAs testing workflow steps (i.e., positive/negative classification, patterns intensity classification, mitotic cell detection, and HEp-2 cell patterns identification) could be integrated into a single practical end-to-end HEp-2 CAD system.

Moreover, due to the high variability of different HEp-2 cell patterns and the limitation of accurately annotating a large number of microscopic images, it is significant to provide an uncertainty quantification (UQ) measure for the DL detectors’ predictions for a robust diagnosis [82,83]. Improving AI models to produce quantitative uncertainty estimates as additional information to their predictions is important to alert human experts about difficult or unknown prediction cases, which is critical for building reliable medical diagnosis systems [84,85]. Integrating such a UQ mechanism with the HEp-2 detection DL models are supposed to provide a real-world reliable HEp-2 CAD system that supports decision-making for autoimmune disease diagnosis.

## 5. Conclusions

Mitotic cell detection from the whole HEp-2 specimen images is a mandatory procedure to approve the correctness of the ANAs slide preparation and to support accurate classification for other interphase cell patterns. This paper presents an automatic deep learning framework to detect the mitotic cells directly from the microscopic HEp-2 specimen images. Due to the scarcity of the labeled dataset, the deep active learning (DAL) approach is involved in annotating the mitotic cells supporting the supervised deep learning models to achieve better detection performance. The experimental results demonstrated the superiority of the YOLO-based framework over the Faster R-CNN counterpart in this task. Data pre-processing via CLAHE improved the prediction performance of both YOLO and Faster R-CNN detectors by mAP of 4% and 7%, respectively. Moreover, the employed data augmentation techniques boosted the detection performance of the proposed HEp-2 mitotic cell detection framework by mAP of 7.5% and 8.3% for YOLO and Faster R-CNN, respectively. The proposed framework shows its capability and reliability to automatically detect multiple mitotic cells from diverse types of HEp-2 specimen images.

## Figures and Tables

**Table 1:** I3A Task-2 dataset description. Classes annotated as Centromere (Ce), Golgi (Gl), Homogeneous (Ho), Nucleolar (Nu), Nuclear Membrane (Num), Speckled (Sp), and Mitotic Spindle (MS).

Cell Phase	Interphase						Mitotic
**HEp-2 Class**	Ce	Gl	Ho	Nu	NuM	Sp	MS
**No. of specimen images**	200	84	212	204	40	208	60

**Table 2:** Mitotic cell detection evaluation performance (%) using YOLO and Faster R-CNN detectors over a 5-fold testing protocol using the I3A Task-2 Dataset.

Fold Test	YOLO Detector			Faster R-CNN Detector		
	Recall	Precision	mAP	Recall	Precision	mAP
**Fold-1**	89.126	87.147	80.269	86.101	84.122	77.244
**Fold-2**	91.026	89.025	82.652	88.001	86	79.627
**Fold-3**	90.235	88.845	81.345	87.21	85.82	78.32
**Fold-4**	89.542	87.865	81.026	86.517	84.84	78.001
**Fold-5**	90.125	88.652	82.365	87.101	85.627	79.34
**Average**	**90.011**	**88.307**	**81.531**	**86.986**	**85.282**	**78.506**
**SD**	** ±0.72 **	** ±0.78 **	** ±0.98 **	** ±0.72 **	** ±0.79 **	** ±0.97 **

**Table 3:** Execution time comparison of the adopted deep learning detection algorithms.

Deep Learning Detector	Dataset	Training Time/Epoch (Second)	Testing Time/a Single Specimen Image (ms)	FPS
**YOLO**	I3A Task-2	600	180	5.56
**Faster** **R-CNN**		1225	330	3.03

**Table 4:** Comparison between the proposed AI-based detection framework with other recent works. In table: BcA is the Balance class Accuracy.

Reference	Framework Description				vBcA	mAP@0.5
	Dataset	Task Definition	Features Extraction	Classifier		
Tonti et al. (2015) [31]	Subset derived from MIVIA dataset		Morphological rules for selecting mitotic group + GLCM [32] for classification	Unsupervised K-means clustering	0.82	-
Miros et al. (2015) [11]	Subset derived from I3A dataset (cell-level images)		Combination of Shape (elliptical, roundness, etc.), size, intensity (i.e., histogram, mean, etc.) and texture (GLCM [32])	SVM	0.72	-
Gupta et al. (2019) [35]		Binary classification (mitotic vs. interphase)	CNN- based feature (using AlexNet [76]) +Decision-level fusion + BoW [77]	One-Class SVM	0.95	-
Gupta et al. (2019) [36]	I3A Task-3 dataset (cell-level images)		Siamese CNN- based feature (trained with triplet-loss [78])	SVM	0.82	-
Gupta et al. (2020) [37]			CNN-based feature (using AlexNet [76]) + BoW [77] + LM filter bank [79] + DCGAN [60] augmentation	SVM	0.98	-
Gupta et al. (2020) [17]	I3A Task-2 (Specimen images)	Detection of Mitotic spindle specimen type	Faster R-CNN [16]		0.98	-
**Proposed** **Framework** *	**I3A Task-2** **(Specimen images** **(annotated using** **DAL)**	**Detection of** **mitotic cells from** **specimen images**	**End-to-end deep** **detection model**		**Recall: 0.90** **Precission:0.88**	**0.815**

* The best evaluation results that derived using the proposed AI framework based on the YOLO predictor.

## Data Availability

To achieve this study, free public “I3A Task-2 HEp-2 specimen” dataset is used. Weblink: https://hep2.unisa.it/dbtools.htmls (accessed on March 2023).

## Abbreviations

The following abbreviations are used in this manuscript:

IIF	Indirect Immunofluorescence
HEp-2	Human Epithelial type 2 Cells
ANAs	Anti-Nuclear Antibodies
CTD	Connective Tissue Diseases
AI	Artificial intelligence
CAD	Computer-Aided Detection
FITC	Fluorescein-isothiocyanate imaging channel
DAPI	The 4’,6-diamidino-2-phenylindole imaging channel
GT	ground truth labeling
DAL	Deep Active Learning
CLAHE	contrast-limited adaptive histogram equalization
MES	Multi-expert system
GLCM	Gray-Level Co-occurrence Matrix
DCGAN	Deep Convolutional Generative Adversarial Networks
CNN	Convolutional Neural Network
SSIM	Structural Similarity Index
RPN	Region Proposal Network
RoI	Region of Interest
NMS	Non-maximum Suppression
SGD	Stochastic Gradient Descent
mAP	Mean Average Precession
IoU	Intersection over Union
YOLO	You Only Look Once
TP	True Positive
FP	False Positive
FN	False Negative
UQ	Uncertainty Quantification
